# Natural Killer Cell and Extracellular Vesicle-Based Immunotherapy in Thyroid Cancer: Advances, Challenges, and Future Perspectives

**DOI:** 10.3390/cells14141087

**Published:** 2025-07-16

**Authors:** Kruthika Prakash, Ramya Lakshmi Rajendran, Sanjana Dhayalan, Prakash Gangadaran, Byeong-Cheol Ahn, Kandasamy Nagarajan Aruljothi

**Affiliations:** 1 Department of Genetic Engineering, SRM Institute of Science and Technology, Kattankulathur, Chengalpattu 603203, India; kp8418@srmist.edu.in (K.P.); sd2312@srmist.edu.in (S.D.); 2 Department of Nuclear Medicine, School of Medicine, Kyungpook National University, Daegu 41944, Republic of Korea; ramyag@knu.ac.kr; 3 Cardiovascular Research Institute, Kyungpook National University, Daegu 41944, Republic of Korea; 4 BK21 FOUR KNU Convergence Educational Program of Biomedical Sciences for Creative Future Talents, Department of Biomedical Sciences, School of Medicine, Kyungpook National University, Daegu 41944, Republic of Korea; 5 Department of Nuclear Medicine, Kyungpook National University Hospital, Daegu 41944, Republic of Korea

**Keywords:** thyroid cancer, natural killer (NK) cells, tumor microenvironment, immunotherapy, CAR-NK cells

## Abstract

Thyroid cancer, the most frequently occurring endocrine neoplasm, comprises a heterogeneous group of histological subtypes, spanning from the indolent papillary thyroid carcinoma (PTC) to the rapidly progressive and lethal anaplastic thyroid carcinoma (ATC). Although conventional therapies, such as surgery and radioactive iodine (RAI), are effective for differentiated thyroid cancers, treatment resistance and poor prognosis remain major challenges in advanced and undifferentiated forms. In current times, growing attention has been directed toward the potential of Natural Killer (NK) cells as a promising immunotherapeutic avenue. These innate immune cells are capable of direct cytotoxicity against tumor cells, but their efficiency is frequently compromised by the immunosuppressive tumor microenvironment (TME), which inhibits NK cell activation, infiltration, and persistence. This review explores the dynamic interaction between NK cells and the TME in thyroid cancer, detailing key mechanisms of immune evasion, including the impact of suppressive cytokines, altered chemokine landscapes, and inhibitory ligand expression. We further discuss latest advancements in NK cell-based immunotherapies, including strategies for ex vivo expansion, genetic modification, and combinatorial approaches with checkpoint inhibitors or cytokines. Additionally, emerging modalities, such as NK cell-derived extracellular vesicles, are addressed. By combining mechanistic insights with advancing therapeutic techniques, this review provides a comprehensive perspective on NK cell-based interventions and their future potential in improving outcomes for patients with thyroid cancer.

## 1. Introduction

Cancer remains a predominant cause of morbidity and mortality globally, leading to nearly one in six deaths globally. Despite significant advancements in diagnosis and treatment, the global cancer burden continues to rise, driven by aging populations, environmental factors, and lifestyle changes [1]. Among various malignancies, thyroid cancer represents the predominant endocrine tumor, with its prevalence increasing steadily over recent decades. According to GLOBOCAN 2020, thyroid cancer accounts for approximately 586,000 new cases and over 43,000 deaths annually, ranking ninth among the most frequently diagnosed cancers [2].

Thyroid cancer comprises several histological subtypes, including papillary thyroid carcinoma (PTC), follicular thyroid carcinoma (FTC), medullary thyroid carcinoma (MTC), and anaplastic thyroid carcinoma (ATC). Although differentiated thyroid cancers, such as PTC and FTC, often exhibit good prognosis with standard therapies, approximately 5–15% of cases eventually dedifferentiate or become refractory to radioactive iodine (RAI) therapy, leading to treatment resistance and poor outcomes [3,4]. In particular, aggressive variants, such as ATC and advanced MTC, are associated with poor survival and intrinsic resistance to conventional treatments. This disparity underscores the pressing need for more effective, targeted, and immune-based therapeutic strategies [5].

Natural Killer (NK) cells, integral effectors of the innate immune system, are increasingly recognized to have potent anti-tumor activity [6,7]. Unlike T cells, NK cells exhibit the capacity to identify and eliminate cancer cells without prior sensitization, making them attractive candidates for immunotherapy [8]. However, in thyroid cancer, especially in aggressive forms such as ATC and MTC, the tumor microenvironment (TME) poses significant immunosuppressive barriers. Cytokines and soluble mediators, namely transforming growth factor-β (TGF-β), interleukin-10 (IL-10), indoleamine 2,3-dioxygenase (IDO), and prostaglandin E2 (PGE2), can impair NK cell infiltration, downregulate activating receptors, and suppress cytotoxic activity [9].

In light of these challenges, recent advances in NK cell-based immunotherapy offer promising avenues for intervention. These include novel strategies for NK cell expansion and activation, genetic engineering, combination therapies with checkpoint inhibitors, and the use of extracellular vesicles generated from NK cells. Moreover, adoptive transfer of allogeneic or stem cell-derived NK cells is gaining momentum, with several clinical trials assessing their safety and efficiency in solid tumors, including thyroid cancer [10,11]. This review intends to analyze the dynamic function of NK cells in thyroid cancer, elucidate mechanisms of immune evasion within the TME, and critically examine current and emerging NK cell-based immunotherapeutic approaches, along with their limitations and future potential.

## 2. NK Cell Biology and Their Role in Thyroid Cancer

NK cells are important elements of the innate immune system, liable for the early identification as well as elimination of virally infected or malignant cells. Originating from common lymphoid progenitors in the bone marrow, human NK cells are distinguished by the presence of CD56 and the absence of CD3. They are broadly classified into CD56^bright^ cells, recognized for cytokine production, and CD56^dim^ cells, which exhibit potent cytotoxicity through the release of perforin, granzymes, and engagement of death receptor pathways [12].

The activity of NK cells is meticulously controlled by a balance of inhibitory and activating signals. Activating receptors, such as NK group 2 member D (NKG2D), NK cell p30-related protein (NKp30), NK cell p44-related protein (NKp44), NK cell p46-related protein (NKp46), and DNAX accessory molecule-1 (DNAM-1), recognize stress-induced ligands on infected or malignant cells, promoting cytotoxic responses. Inhibitory receptors, such as NKG2A and killer-cell immunoglobulin-like receptors (KIRs), bind to self-major histocompatibility complex (MHC) class I molecules, suppressing NK cell activation to protect healthy tissue. NK cells can detect this loss of “self” termed the “missing-self” response and selectively eliminate such MHC-I-deficient cells, highlighting their role in tumor immunosurveillance [13].

Thyroid cancer includes well-differentiated types such as PTC and FTC, as well as more aggressive forms such as MTC and ATC. Although PTC and FTC are typically immunologically “cold” with limited immune cell infiltration, dedifferentiated forms such as ATC exhibit a highly immunosuppressive TME that further dampens NK cell activity [14].

The TME in thyroid cancer plays a pivotal role in excluding or inactivating NK cells. Tumor-derived soluble factors, such as PGE2, TGF-β, vascular endothelial growth factor (VEGF), and IL-10, are known to suppress NK cell activity. These immunosuppressive molecules downregulate the expression of principal activating receptors on NK cells, namely NKG2D, NKp30, and NKp46, and impair the synthesis of effector cytokines such as interferon-gamma (IFN-γ) [15]. Among these, PGE2 is particularly significant in thyroid cancer. It is secreted by tumor cells and acts via the E-type prostanoid receptor 4 (EP4) on NK cells to inhibit their maturation, reduce the expression of cytotoxic molecules such as granzyme B and perforin, and limit their ability to lyse tumor targets [16].

Another immunosuppressive mechanism in thyroid cancer involves indoleamine 2,3-dioxygenase enzyme, which degrades tryptophan into immunosuppressive metabolites. This tryptophan depletion and metabolite accumulation create a hostile environment for NK cells, reducing the expression of activating receptors such as NKp46 and NKG2D, impairing cytotoxicity as well as cytokine secretion [17].

In PTC, tumor cells secrete chemokines that attract immune-suppressive cells, namely myeloid-derived suppressor cells (MDSCs) and regulatory T cells (Tregs), rather than NK cells. These cells suppress NK cell activity and block their infiltration. In contrast, ATC tumor cells upregulate stress ligands, such as UL16-binding proteins (ULBPs) and MHC class I polypeptide-related sequence A/B (MICA/B), which are ligands for NKG2D on NK cells, suggesting susceptibility to NK-mediated killing [18]. However, ATC also shows high programmed death-ligand 1 (PD-L1) expression and secretes cytokines such as IL-6 and IL-10, contributing to an immunosuppressive TME [19]. IL-6, in particular, promotes the expansion of regulatory T cells, dampens cytotoxic T cell responses, and, along with IL-10, contributes to NK cell exhaustion [20]. Furthermore, ATC is associated with the upregulation of several immune checkpoint molecules, including T cell immunoreceptor with Ig and ITIM domains (TIGIT), T cell immunoglobulin and mucin-domain containing-3 (TIM-3), and programmed cell death protein 1 (PD-1), contributing to immune evasion [21]. MTC, derived from parafollicular C cells, presents a dense stromal environment with limited NK cell infiltration [22]. Although direct studies are limited, it is proposed that the physical barrier and immunosuppressive milieu significantly impair NK cell recruitment and function.

Importantly, the density and activation status of tumor-infiltrating NK cells are correlated with clinical outcomes. Low infiltration or functional impairment is linked to poor prognosis and treatment resistance, whereas higher NK cell presence and activity correlate with better disease control. Beyond direct cytotoxicity, NK cells also regulate adaptive immunity by stimulating dendritic cell maturation through IFN-γ, enhancing T cell responses.

## 3. Advances in NK Cell-Based Immunotherapy for Thyroid Cancer

Despite the immunosuppressive nature of the thyroid tumor microenvironment, recent years have witnessed significant progress in utilizing the potential NK cells for treating thyroid cancer. NK cells offer a distinct advantage in cancer immunotherapy because of their capacity to identify and eliminate tumor cells independent of antigen presentation, a pathway often compromised in thyroid cancer. However, the limited invasion and dysfunction of NK cells in aggressive thyroid cancers have prompted efforts to enhance their activity and persistence. Technological advancements in NK cell expansion, genetic engineering, and adoptive transfer, along with rational combination strategies, are paving the way for innovative and effective NK cell-based therapies in thyroid cancer. The following subsections explore the major approaches being pursued in this area.

### 3.1. NK Cell Expansion and Activation Strategies

**Figure 1:**
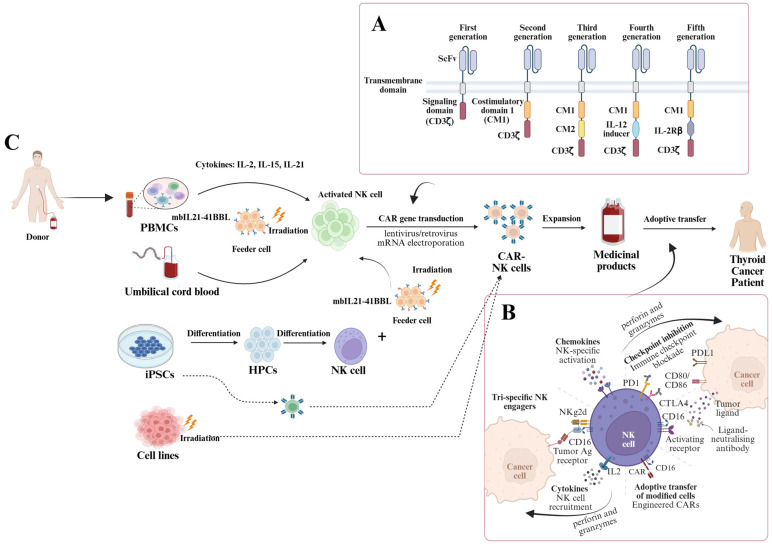
Generation of CAR-NK Cells for Adoptive Immunotherapy in Cancer Patients. (**A**) The structural design of chimeric antigen receptors (CARs) has evolved from first- to fifth-generation constructs, incorporating NK cell-specific intracellular signaling domains. (**B**) CAR-NK cells mediate tumor cell destruction through both CAR-dependent mechanisms and CAR-independent pathways, leveraging their innate immune properties. (**C**) Various cellular sources are employed for the generation of CAR-NK cell products, each with specific protocols for expansion, genetic modification, and clinical-grade manufacturing. CAR: Chimeric Antigen Receptor; NK cell: Natural Killer cell; PBMCs: Peripheral Blood Mononuclear Cells; iPSCs: Induced Pluripotent Stem Cells; HPCs: Hematopoietic Progenitor Cells; mbIL21-41BBL: Membrane-bound Interleukin-21 and 4-1BB Ligand; IL-2: Interleukin-2; IL-15: Interleukin-15; IL-21: Interleukin-21; CD3ζ: Cluster of Differentiation 3 zeta chain; CM1: Costimulatory Molecule 1; CM2: Costimulatory Molecule 2; IL-2Rβ: Interleukin-2 Receptor Beta; ScFv: Single-chain Variable Fragment; PD-L1: Programmed Death-Ligand 1; PD1: Programmed Cell Death Protein 1; CTLA4: Cytotoxic T-Lymphocyte-Associated Protein 4; LAG3: Lymphocyte Activation Gene 3; TIGIT: T Cell Immunoreceptor with Ig and ITIM Domains; Ag: Antigen. Created in https://BioRender.com.

One of the foremost challenges in NK cell-based immunotherapy is acquiring a sufficient quantity of functional NK cells for therapeutic use. NK cells constitute a small fraction of peripheral blood lymphocytes, and their ex vivo expansion without losing cytotoxic potential is essential to harness their therapeutic potential (Figure 1).

Cytokines such as IL-21, IL-2, and IL-15 have been commonly used to stimulate and expand NK cells. Among them, IL-15 is particularly effective, promoting NK cell proliferation and survival without inducing Tregs [23]. Furthermore, memory-like NK cells, generated by preactivation with IL-12, IL-15, and IL-18, show superior persistence and cytotoxicity compared to conventional NK cells. These cytokine-induced memory-like (CIML) NK cells have been shown to overcome TME-induced dysfunction in other solid tumors and may hold promise in thyroid cancer as well [24].

Feeder cells, such as K562 cells genetically altered to express membrane-bound IL-21 or IL-15 and 4-1BB ligand (K562-mbIL21-41BBL), have been employed to achieve robust NK cell expansion. These systems can yield a large number of highly cytotoxic NK cells within 2–3 weeks [25]. Feeder cell-expanded NK cells have been shown to exhibit enhanced degranulation and IFN-γ production, which are key in-dicators of antitumor activity. Although this approach has been widely studied in hematological malignancies, emerging preclinical models of thyroid cancer are beginning to incorporate these strategies [26].

To standardize expansion protocols, artificial antigen-presenting cells (aAPCs) coated with co-stimulatory ligands and cytokines have been developed. aAPC systems allow for standardized, GMP (Good Manufacturing Practices)-compliant expansion, critical for clinical translation. In a recent pilot study demonstrated that aAPC-expanded NK cells exhibited enhanced cytotoxic activity against thyroid cancer spheroids in a 3D culture model, simulating a more realistic TME [27].

Bioreactors such as G-Rex and Wave systems provide scalable, clinically compliant environments for NK cell production. These platforms enhance oxygenation and nutrient exchange, supporting high-density NK cell cultures with preserved functionality. G-Rex flasks have demonstrated up to 1000-fold NK cell expansion over two weeks with preserved cytotoxic activity [28]. Wave bioreactors provide dynamic culture conditions that mimic physiological flow and shear stress, contributing to improved NK cell activation and viability.

Another promising avenue involves the generation of NK cells from umbilical cord blood (UCB)-derived CD34^+^ hematopoietic stem cells or induced pluripotent stem cells (iPSCs). This approach allows for the production of uniform, off-the-shelf NK cell products, which is particularly valuable for immunotherapy against heterogeneous tumors such as ATC [29]. Stem cell-derived NK cells can be genetically engineered or pre-activated during differentiation to enhance cytotoxicity and persistence. Studies have shown that iPSC-derived NK cells express activating receptors, such as NKG2D and DNAM-1, and are capable of lysing thyroid tumor cells in vitro [30]. iPSC-derived NK cells can also be engineered with chimeric antigen receptors (CARs), enhancing specificity and expanding therapeutic applications in aggressive thyroid cancers.

### 3.2. Genetically Engineered NK Cells

Genetic engineering of NK cells represents a transformative strategy to enhance their tumor-targeting capabilities, persistence, and resistance to the immunosuppressive TME, particularly in challenging solid tumors such as thyroid cancer.

A highly promising strategy in NK cell therapy is the use of CAR-NK cells, which are specially modified to carry artificial receptors. These receptors help the NK cells to better recognize tumor-associated antigens (TAAs) with high specificity. Schiff et al. (2021) demonstrated that NK-92 cells engineered to express a CAR targeting epidermal growth factor receptor (EGFR) exhibited potent cytotoxic effects against ATC cell lines in vitro and significantly reduced tumor growth in xenograft models [31].

CAR-NK cells specifically engineered to recognize mucin 1 (MUC1) have demonstrated improved cytotoxicity against ana-plastic thyroid carcinoma (ATC) cells and produced higher levels of IFN-γ, a key molecule that enhances the immune response against tumors [32]. Similarly, CAR-NK cells targeting PD-L1 were able to effectively kill ATC and MTC cells, which typically express high levels of PD-L1, demonstrating their potential to overcome tumor immune evasion [33].

Beyond TAAs, genetic modifications have also focused on improving NK cell activation and persistence. Enhancing NK cell persistence has been achieved through the expression of membrane-bound cytokines such as IL-15 or IL-21. Silvestre et al. (2023) reported that membrane-bound IL-15 (mbIL-15) expressing NK cells demonstrated increased tumor infiltration and cytotoxicity even under cytokine-deprived conditions, which is particularly relevant in thyroid tumors [34]. Building on this, NK cells were engineered with dual IL-15/IL-21 expression, specifically targeting RET-mutant MTC. These dual-cytokine armed NK cells displayed superior anti-tumor efficacy both in vitro and in vivo, delaying tumor progression in murine models [35]. Moreover, CRISPR/Cas9 gene editing has enabled the targeted knockout of inhibitory molecules, such as PD-1 and cytokine-inducible SH2-containing protein, both of which negatively regulate NK cell activation. CRISPR editing was applied to enhance NK cell responsiveness in thyroid tumor organoid systems, laying the groundwork for future gene-edited NK therapies in thyroid cancer [36].

Finally, engineering NK cells to express high-affinity CD16 variants (FcγRIIIa) has shown promise in boosting antibody-dependent cellular cytotoxicity (ADCC) when combined with monoclonal antibodies targeting EGFR or VEGF, both of which are upregulated in thyroid cancer [37]. Modified NK cells also exhibited superior tumor cell killing when co-administered with targeted antibodies, underscoring the synergistic potential of combining engineered NK cells with existing biologics [38].

### 3.3. Combination Therapies

Although NK cell-based therapies have shown potential against thyroid cancers, particularly aggressive subtypes such as ATC, their efficacy is often limited by the immunosuppressive TME. Rational combination strategies aim to overcome these barriers, enhance NK cell function, and improve clinical outcomes (Table 1).

Checkpoint blockade inhibitors are among the most widely explored combination partners for NK cell therapy. Immune checkpoints, namely PD-1/PD-L1, TIGIT, and NKG2A, are often upregulated in thyroid tumors, leading to NK cell exhaustion and reduced cytotoxicity [46]. Research has demonstrated that amalgamating NK cells with anti-PD-1 or anti-PD-L1 antibodies restores NK function and enhances anti-tumor responses [19]. In a preclinical model of ATC, combination therapy involving PD-L1 blockade and NK cell-based immunotherapy significantly augmented the cytolytic efficacy of NK cells against tumor spheroids and resulted in a marked decrease in tumor load in vivo [47]. Similarly, TIGIT blockade in combination with ex vivo activated NK cells improved cytokine secretion and target cell killing in PTC models, suggesting that checkpoint inhibition could reinvigorate NK cell responses even in otherwise suppressive environments [48].

Additionally, radiotherapy and targeted small-molecule inhibitors have been demonstrated to synergize with NK cell therapy. Sub-lethal doses of radiation can induce immunogenic modulation in tumor cells via enhancing the expression of NK cell activating ligands such as MICA/B and ULBP1-3, thereby enhancing NK-mediated killing [49]. In thyroid cancer, radiation has been documented to enhance the expression of NKG2D ligands on tumor cells, hence increasing their vulnerability to NK cell recognition [50,51]. Targeted agents such as lenvatinib modulate the TME by reducing VEGF-driven suppression. When combined with NK cells, lenvatinib improved vascular normalization and survival in thyroid cancer models [52].

Cytokine adjuvants, particularly IL-21, IL-15, and IL-2, are essential for NK cell proliferation but are limited by systemic toxicity. Localized delivery or engineering NK cells with membrane-bound IL-15 offers a solution [53,54]. Studies have demonstrated that mbIL-15 expressing NK cells combined with low-dose IL-21 led to sustained expansion and improved tumor clearance in MTC models. IL-15 superagonists such as ALT-803 also enhance NK cell function without severe cytokine release syndrome [42,55].

### 3.4. Adoptive NK Cell Therapy and Clinical Trials

Adoptive NK cell therapy entails the infusion of enlarged autologous or allogeneic NK cells that are activated ex vivo to target cancer cells. This approach has demonstrated potential outcomes in hematologic malignancies, and its application in solid tumors, including thyroid cancers, is gaining traction due to advances in expansion protocols, genetic engineering, and combination regimens [56,57] (Table 2).

Aggressive subtypes, such as ATC and RAI-refractory differentiated thyroid cancer (RAI-R DTC), are potential candidates due to their resistance to conventional therapies. Preclinical studies have demonstrated that ex vivo-activated NK cells can efficiently lyse ATC cells in vitro and decrease tumor proliferation in murine xenograft models [7]. Wennerberg et al. (2014) showed that ATC cells expressing high levels of NKG2D ligands are particularly susceptible to NK cell-mediated cytotoxicity when NK cells are adequately activated [50].

A notable clinical advancement is the use of cytokine-induced CIML NK cells, which are pre-activated with IL-18, IL-15, and IL-12 before infusion. These cells exhibit enhanced persistence, cytotoxicity, and cytokine secretion. Although primarily evaluated in hematologic cancers, early-phase trials are expanding their use in solid tumors, including thyroid malignancies [24].

UCB-derived NK cells have also entered clinical exploration. These allogeneic NK cells are attractive due to their reduced risk of graft-versus-host disease (GVHD) and their availability as off-the-shelf products [58]. A phase I trial (NCT04319768) demonstrated their safety and early clinical activity in individuals with advanced solid neoplasms, including thyroid carcinoma.

Moreover, CAR-NK cells targeting thyroid-specific antigens are under preclinical development. Studies have shown that anti-CD70 CAR-NK cells effectively lyse ATC cells both in vitro and in vivo and maintain activity in immunosuppressive conditions [56]. Importantly, these engineered NK cells maintain reduced risk of cytokine release syndrome and neurotoxicity in comparison to CAR-T cells, making them attractive for clinical application [59].

Combination strategies are increasingly being tested in clinical trials. For instance, NCT05036016 is evaluating adoptive NK cell infusion with lenvatinib in RAI-R DTC and ATC patients to assess synergy and immune modulation.

**Table 2:** This table presents a summary of preclinical and clinical studies involving NK cell-based therapies in thyroid cancer.

Study	NK Cell Type/Approach	Study Type/Model	Key Findings	Relevance to Thyroid Cancer	Ref
Activated NK cells	Ex vivo–activated autologous NK cells	Preclinical (in vitro ATC cells, murine xenograft model)	NK cells lysed ATC cells expressing high NKG2D ligands; reduced tumor proliferation	Demonstrates cytotoxic efficiency of activated NK cells against ATC	[60]
CIML NK Cells	IL-12, IL-15, and IL-18 pre-activated NK cells	Early-phase clinical trials (hematologic malignancies); expansion into solid tumors ongoing	Enhanced persistence, cytokine secretion, and cytotoxicity	Being explored in thyroid malignancies as next-generation NK therapy	[24]
UCB-derived NK cells	Allogeneic NK cells derived from umbilical cord blood	Clinical Trial, Phase I (NCT04319768)	Demonstrated safety and early anti-tumor activity	Safe, off-the-shelf product being tested in solid tumors, including thyroid carcinoma	[61]
Anti-CD70 CAR-NK cells	Genetically engineered NK cells targeting CD70	Preclinical (in vitro ATC cells, mouse xenograft model)	Effective tumor cell lysis under immunosuppressive conditions; low CRS/neurotoxicity risk	Promising preclinical candidate for ATC treatment	[59]
Lenvatinib + NK cell	Combination of targeted therapy and adoptive NK cells	Clinical Trial, Phase I/II (NCT05036016)	Evaluating synergy and immune modulation in RAI-R DTC and ATC	Ongoing trial combining NK therapy with standard-of-care TKI	[62]

NK cells: Natural Killer cells; ATC: Anaplastic Thyroid Carcinoma; NKG2D: NK group 2 member D; CIML NK Cells: Cytokine-Induced Memory-like Natural Killer cells; IL-12: Interleukin-12; IL-15: Interleukin-15; IL-18: Interleukin-18; UCB: Umbilical Cord Blood; CAR: Chimeric Antigen Receptor; CD70: Cluster of Differentiation 70; CRS: Cytokine Release Syndrome.

## 4. NK Cell-Derived Extracellular Vesicles

**Figure 2:**
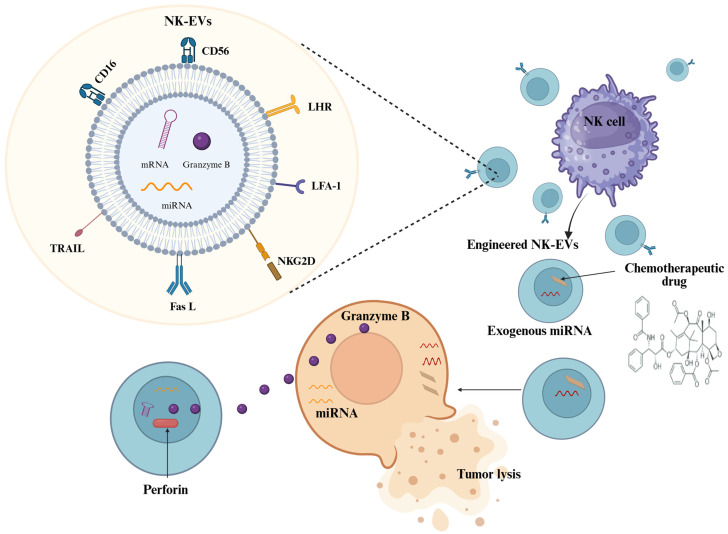
Schematic representation of NK cell-derived extracellular vesicles. NK-EVs carrying cytotoxic proteins, miRNAs, and surface ligands that mediate antitumor effects. These EVs interact with tumor cells, inducing apoptosis and modulating gene expression. CD16: Cluster of Differentiation 16; CD56: Cluster of Differentiation 56; Fas L: Fas Ligand; LFA-1: Lymphocyte Function-associated Antigen 1; LHR: Luteinizing Hormone Receptor; miRNA: MicroRNA; mRNA: Messenger RNA; NKG2D: Natural Killer Group 2D; NK: Natural Killer; NK-EVs: Natural Killer Cell-Derived Extracellular Vesicles; TRAIL: TNF-Related Apoptosis-Inducing Ligand. Created in https://BioRender.com.

NK cell-derived extracellular vesicles (NK-EVs), including exosomes and microvesicles, are nanosized carriers of cytotoxic proteins and regulatory RNAs. These EVs mirror the anti-tumor functions of NK cells but offer additional advantages, such as improved stability, enhanced tumor penetration, and resistance to tumor-induced immunosuppression (Figure 2). This makes them especially promising for solid tumors, such as thyroid cancer, where TME-mediated suppression limits conventional immunotherapies [63].

NK-EVs are loaded with effector molecules, such as perforin, granzyme A and B, TNF-related apoptosis-inducing ligand (TRAIL), and Fas ligand, along with IFN-γ and NKG2D ligands. Once internalized by tumor cells, NK-EVs activate caspase-mediated apoptotic pathways and disrupt mitochondrial integrity, leading to cancer cell death. Importantly, unlike intact NK cells, these vesicles are not hindered by inhibitory signals or hypoxia within the TME [60].

Sorafenib was loaded into NK exosome mimetics (EM) by mixing sorafenib with NK cells during NK-EM production (NK-EM-S) and they were efficiently taken up by thyroid cancer cells. Bioluminescence imaging revealed that NKEM-S significantly inhibited cell proliferation and induced cytotoxicity in two thyroid cancer cell lines. Notably, NK-EM-S demonstrated strong killing effects against anaplastic thyroid cancer cells, suggesting potent therapeutic potential for aggressive thyroid malignancies [64]. Although direct studies in thyroid cancer are limited, extrapolation from other solid tumors is encouraging. ATC cells express stress ligands such as MICA/B, which are recognized by NKG2D. NK-EVs are likely to exert cytotoxic effects. Their potential to bypass TME suppression makes them particularly suitable for thyroid cancers with high PD-L1 expression and inflammatory cytokines [65]. Additionally, NK-EVs can be engineered to deliver therapeutic cargos such as siRNAs, miRNAs, or drugs. This allows for targeted delivery against thyroid-specific markers such as TSHR or EGFR. As off-the-shelf products, NK-EVs are easier to scale and store than cell therapies and pose no risk of GVHD [63].

Challenges remain, including standardizing large-scale production, ensuring tumor targeting, and initiating clinical studies. However, NK-EVs offer a promising, next-generation immunotherapeutic platform for aggressive thyroid cancers resistant to conventional treatments.

## 5. Challenges and Limitations

Despite the substantial therapeutic potential of NK cell-based immunotherapy in thyroid cancer, multiple barriers impede its clinical implementation and sustained efficacy. A major limitation is the highly immunosuppressive TME, particularly in aggressive variants such as ATC. Within this milieu, elevated concentrations of immunoregulatory cytokines, including TGF-β, IL-10, and PGE2, attenuate NK cell functionality by downregulating key activating receptors such as NKG2D and NKp30, and by impairing the production of effector cytokines [66].

Another limitation is the inefficient trafficking and infiltration of NK cells into solid tumors due to the poor expression of chemokine receptors that match tumor-secreted chemokines. For example, in PTC, the chemokine profile tends to favor the recruitment of immunosuppressive cells, such as Treg cells and MDSCs, which supersedes that of NK cells, thereby diminishing the efficacy of adoptively transplanted NK cells [18].

Furthermore, NK cells exhibit short in vivo persistence and limited proliferation compared to T cells, reducing their long-term therapeutic impact. Although strategies, such as IL-15 engineering and CAR modification, are being explored to enhance persistence and specificity, these are still in early clinical phases and face safety, regulatory, and cost-related challenges [61]. Additionally, the variability in NK cell sources, such as donor-dependent heterogeneity in peripheral blood-derived NK cells or the experimental nature of UCB- or iPSC-derived NK cells, complicates standardization [67].

The large-scale, GMP-compliant production of NK cells or their EVs also remains technically demanding and cost-intensive. Furthermore, CAR-NK cell development specific to thyroid cancer is still in its infancy, with a lack of reliable preclinical models that mimic the thyroid tumor microenvironment, and safety concerns such as off-target effects and cytokine release syndrome remain unresolved [68]. Most critically, there is a lack of robust clinical trials investigating NK cell-based therapies for thyroid cancer, largely due to the relatively low incidence and indolent progression of many thyroid cancers, resulting in limited pharmaceutical interest and scarce translational data [69].

Another emerging concern relates to the long-term safety of checkpoint blockade strategies. Although targeting inhibitory receptors such as PD-1, TIGIT, or NKG2A can reinvigorate exhausted NK or T cells, these molecules play critical roles in maintaining immune tolerance and preventing tissue damage under physiological conditions [70]. Prolonged survival or hyperactivation of cytotoxic lymphocytes lacking inhibitory checkpoints may increase the risk of off-target effects, including autoimmunity, systemic inflammation, or damage to normal tissues [71,72]. Therefore, although promising, checkpoint disruption approaches must be carefully evaluated for safety and reversibility, especially in the context of chronic or indolent thyroid cancers, where long-term immune regulation is essential.

## 6. Future Perspectives and Emerging Strategies

Future directions in NK cell-based immunotherapy for thyroid cancer focus on improving efficacy, persistence, and specificity. Off-the-shelf NK cell products derived from iPSCs or UCB are gaining attention for their scalability and reduced immunogenicity. Genetic engineering, especially CAR-NK cells targeting thyroid cancer-associated antigens, offers enhanced tumor recognition and cytotoxicity [73]. Combination therapies, such as NK cells with immune checkpoint inhibitors or BRAF-targeted agents, are under investigation to overcome tumor immune evasion [74]. Additionally, NK cell-derived extracellular vesicles provide a novel, cell-free therapeutic platform capable of penetrating the tumor stroma and delivering cytotoxic molecules directly. Advances in bioreactor technology and GMP-compliant manufacturing will further support large-scale, clinical-grade NK cell production. Personalized strategies and real-time monitoring are expected to optimize patient outcomes. These innovations together hold promise for transforming the treatment landscape of aggressive thyroid cancers [63].

## 7. Conclusions

NK cells represent an intriguing frontier in the immunotherapy landscape for thyroid cancer. Despite the inherent challenges posed by the immunosuppressive TME, including reduced infiltration, downregulation of activating receptors, and cytokine-mediated dysfunction, NK cells have demonstrated significant potential, especially against aggressive thyroid cancer subtypes such as ATC. Advances in NK cell expansion and activation techniques, genetic modifications such as CAR-NK cells, and synergistic combination therapies have opened new avenues for enhancing NK cell efficacy. Furthermore, the emergence of adoptive NK cell transfer, stem cell-derived NK cells, and NK-EVs offers scalable and potentially off-the-shelf therapeutic options. However, clinical translation still faces hurdles, such as persistence, trafficking, and resistance within the TME. Moving forward, integrating biomarker-driven approaches, improving delivery methods, and conducting robust clinical trials will be key to optimizing NK cell-based therapeutic strategies. With continued innovation and collaboration across disciplines, NK cell-based immunotherapy presents considerable potential to transform the treatment paradigm in thyroid cancer.

## Figures and Tables

**Table 1:** This table presents an overview of current and emerging combination therapies integrating Natural Killer (NK) cell-based immunotherapy with other treatment modalities for thyroid cancer.

Combination Strategy	Agents Used	Rationale	Observed Effects	Relevance to Thyroid Cancer	Study Type/Model/Approval Status	Ref
NK cells + Tyrosine Kinase Inhibitors (TKIs)	NK cells + Sorafenib/Lenvatinib	TKIs reduce tumor-induced immunosuppression and enhance NK cell activity	Enhanced NK cytotoxicity and tumor suppression in vitro and in vivo	Lenvatinib is used in advanced thyroid cancer; synergistic effects expected	Approved therapy; FDA approved in 2015 for RAI-R DTC	[39]
NK cells + Immune Checkpoint Inhibitors	NK cells + Anti-PD-1/Anti-NKG2A	Blocks inhibitory signals that suppress NK function	Increased IFN-γ release and tumor cell lysis	PD-1 and PD-L1 expression noted in thyroid cancer	Clinical trial (NCT02643550—Phase I/II)	[33]
NK cells + Radiotherapy	Radiotherapy + NK cells	Radiation increases expression of stress ligands on tumor cells	Enhanced NK cell recognition and killing	Useful in anaplastic thyroid carcinoma (ATC)	Preclinical study (mouse models of ATC)	[40]
NK cells + Oncolytic Viruses	Oncolytic virus (e.g., HSV) + NK cells	Viral therapy lyses tumor cells and promotes immune infiltration	Enhanced NK recruitment and cytotoxicity	Oncolytic virotherapy is under investigation for thyroid tumors	Preclinical study; HSV-based oncolytics in trials (T-VEC in melanoma, FDA approved 2015)	[41]
NK cells + Cytokine Therapy	IL-15 superagonist (ALT-803) + NK cells	Promotes NK survival, expansion, and function	Sustained NK activity and tumor clearance	Can augment NK therapy in thyroid cancer	Clinical trials (NCT03019666—Phase I/II)	[42]
NK cells + Bispecific Antibodies	NK cells + BiKEs (e.g., CD16xEpCAM)	Directs NK cells to tumor-specific antigens	Improved tumor targeting and cytotoxicity	EpCAM overexpressed in thyroid cancers	Preclinical (in vitro)	[43]
NK cells + TLR Agonists	NK cells + CpG-ODN or Poly(I:C)	Stimulates innate immune signaling and NK activation	Enhanced cytokine secretion and tumor control	Toll-like receptor signaling may synergize in thyroid cancer	Preclinical (in vitro + mouse models)	[44]
NK cells + Small Molecule Inhibitors	NK cells + HDAC inhibitors or PI3K inhibitors	Alters tumor immunogenicity and reverses immune evasion	Restoration of NK sensitivity and tumor suppression	HDACi have shown effects in thyroid tumor models	Preclinical (in vitro and in vivo); HDACi in early-phase trials in thyroid cancer	[45]

NK cells: Natural Killer cells; TKIs: Tyrosine Kinase Inhibitors; PD-1: Programmed Cell Death Protein 1; PD-L1: Programmed Death-Ligand 1; NKG2A: Natural Killer Group 2 Member A; RAI-R DTC: RAI-refractory differentiated thyroid cancer; ATC: Anaplastic Thyroid Carcinoma; HSV: Herpes Simplex Virus; IL-15: Interleukin-15; ALT-803: IL-15 Superagonist Complex; BiKEs: Bispecific Killer Engagers; CD16: Cluster of Differentiation 16; EpCAM: Epithelial Cell Adhesion Molecule; TLR: Toll-Like Receptor; CpG-ODN: Cytosine–phosphate–Guanine Oligodeoxynucleotide; Poly(I:C): Polyinosinic:polycytidylic acid; HDACi: Histone Deacetylase Inhibitors; PI3K: Phosphoinositide 3-Kinases; IFN-γ: Interferon-gamma.

## Data Availability

No data were used for the research described in this article.
